# Artificial Intelligence Readiness in Nursing: A Scoping Review of Knowledge, Attitudes, Practice, and Emerging Evidence on Patient Safety Culture

**DOI:** 10.3390/nursrep16100370

**Published:** 2026-10-19

**Authors:** Sarah Hassan Tawhari, Regie Buenafe Tumala, Yahya Hassan Tawhari

**Affiliations:** 1 College of Nursing, King Saud University, Riyadh 12372, Saudi Arabia; ytohari@moh.gov.sa; 2 Department of Intensive Care Unit, Abu Arish General Hospital, Jazan Health Cluster, Abu Arish 45911, Saudi Arabia; 3 Department of Male General Ward, Abu Arish General Hospital, Jazan Health Cluster, Abu Arish 45911, Saudi Arabia

**Keywords:** artificial intelligence, attitudes, knowledge, nurses, patient safety culture, practice

## Abstract

**Background**: Artificial intelligence (AI) is increasingly integrated into nursing practice, yet nurses often demonstrate low to moderate AI knowledge, mixed attitudes, and inconsistent practice behaviors. Although individual studies have examined these domains, no review has comprehensively examined AI-related knowledge, attitudes, and practice (KAP) together with the emerging evidence on patient safety culture among nurses. This gap is significant because safe AI adoption in nursing depends on both technical competence and a strong safety culture across diverse clinical settings. **Objective**: To systematically map international evidence on nurses’, nursing students’, and other included participants’ AI-related KAP, and to examine how these factors may relate to patient safety culture. Because several eligible studies included multidisciplinary samples in which nurse-specific quantitative data were not reported separately (China, Bangladesh, Pakistan, and three studies from the Kingdom of Saudi Arabia [KSA]), only studies with extractable nurse-specific results were used to inform nurse-focused conclusions, and multidisciplinary findings were not attributed solely to nurses. **Methods**: This scoping review followed Arksey and O’Malley’s framework and adhered to the Preferred Reporting Items for Systematic Reviews and Meta-Analyses extension for Scoping Reviews (PRISMA-ScR) guidelines. Searches of PubMed, CINAHL, Scopus, Web of Science, and ScienceDirect (2021–2026) identified studies examining AI-related perceptions among nurses and other healthcare participants when nurse-specific data were reported separately or extractable. Data extraction and narrative synthesis were conducted using Joanna Briggs Institute guidance. **Results**: Ten studies met the inclusion criteria and were included in the final review. Studies from China, Bangladesh, Pakistan, the KSA, Turkey, and the United Arab Emirates showed variable AI knowledge but predominantly positive attitudes. Ethical concerns—privacy, job displacement, and reduced empathy—were common. Younger age, higher education, and prior AI exposure were associated with more favorable perceptions. Nurses across regions emphasized the need for structured AI training. Only one study examined patient safety culture, and it reported that AI knowledge and attitudes significantly predicted patient safety culture, with attitude showing the stronger effect. However, because this evidence comes from a single cross-sectional study conducted in a multidisciplinary sample without nurse-specific results, it is insufficient to establish causal conclusions, and its findings cannot be generalized specifically to nurses. **Conclusions**: Nurses are generally receptive to AI but lack sufficient knowledge, training, and organizational support for safe implementation. Preliminary evidence suggests a positive association between AI-related knowledge, attitudes, and patient safety culture, but this relationship remains uncertain due to the extremely limited evidence base and the reliance on evidence that is not exclusively nurse-specific. Available studies indicate generally favorable attitudes toward AI alongside variable knowledge, limited formal training, and persistent ethical and organizational concerns, with only one eligible cross-sectional study linking these factors to patient safety culture. The conclusion regarding this association should not be attributed exclusively to nurses because the patient safety culture evidence was derived from a multidisciplinary sample. Future research should explore how AI competencies may interact with safety-related behaviors and organizational systems. Strengthening AI training, ethical guidance, and organizational support remains essential for safe adoption.

## 1. Introduction

Artificial intelligence (AI) has rapidly evolved into a defining technological force in contemporary healthcare, reshaping clinical decision-making, workflow efficiency, and patient outcomes across diverse international settings [1,2,3,4]. Modern AI modalities—including machine learning (ML), deep learning, natural language processing (NLP), computer vision, and large language models (LLMs)—now support complex clinical tasks such as risk prediction, early detection of deterioration, automated documentation, and personalized care planning. Bates et al. [5] emphasize that AI represents “a valuable tool that could be used to improve the safety of care,” with demonstrated applications across major patient safety harm domains including adverse drug events, decompensation, pressure injuries, and diagnostic errors.

The global acceleration of AI adoption is driven by its ability to process large volumes of heterogeneous data from electronic health records (EHRs), sensors, imaging, and patient-generated information. As El Arab et al. [6] note, AI tools now provide “timely, actionable insights that inform diagnosis, risk stratification, treatment planning, resource allocation, and patient engagement.” These capabilities are particularly relevant in high-acuity environments where rapid, data-driven decisions are essential for preventing harm.

Nurses—especially registered nurses in critical care—are central to the safe and effective deployment of AI technologies. Critical care nurses operate in data-intensive, high-risk environments requiring continuous monitoring, rapid clinical judgment, and early recognition of deterioration. AI-enabled tools such as predictive analytics, wearable sensors, and automated alert systems have shown promise in enhancing these functions. Al Khatib and Ndiaye [7] highlight that AI “enables real-time monitoring of patients’ vital signs, predicting potential health deteriorations, and providing alerts for immediate intervention,” positioning nurses as key beneficiaries of AI-supported surveillance.

International evidence demonstrates that AI can enhance nursing practice across education, clinical care, workload management, and decision support. For example, AI-driven monitoring systems allow nurses to detect subtle physiological changes earlier than conventional methods, supporting timelier interventions and improved patient outcomes [1]. Similarly, Ruksakulpiwat et al. [8] identify six major AI-supported nursing care functions—risk identification, health assessment, patient classification, improved care delivery, research development, and care planning—illustrating AI’s potential to augment nursing capabilities.

Despite these benefits, the readiness of nurses to adopt AI—and the extent to which their knowledge, attitudes, and practice (KAP) align with safe implementation—remains insufficiently understood. Existing studies reveal substantial variability in nurses’ understanding of AI capabilities, limitations, and risks. Joo et al. [9] found that while nurses recognized AI’s potential to enhance efficiency and care quality, they also expressed concerns about ethical issues, increased workload, and the risk of overreliance on automated systems. One participant in Rony et al. [10] stated: “*As AI enters our domain, critical ethical questions arise, particularly concerning patient privacy and data security.*”

These concerns are especially salient in critical care units, where the consequences of algorithmic errors, alert fatigue, or workflow misalignment can be severe. Yildirim et al. [11] found that intensive care nurses perceived AI as a valuable decision-support tool but stressed the necessity of human oversight and expressed concerns that overreliance on AI could threaten professional autonomy and the nursing role.

Although AI is increasingly embedded in clinical workflows, there is limited evidence linking nurses’ AI-related KAP to broader patient safety culture. Patient safety culture encompasses shared values, norms, and practices that prioritize safe care delivery, open communication, error reporting, and continuous learning. While AI has the potential to strengthen safety culture—through improved risk prediction, standardized documentation, and enhanced situational awareness—this potential can only be realized when nurses possess adequate AI knowledge, maintain balanced attitudes, and integrate AI tools effectively into practice.

However, current research suggests a persistent disconnect in nurses’ AI-related KAP. For example, AI literacy remains inconsistent, limiting nurses’ ability to accurately interpret and apply AI-generated outputs [12]. Attitudes toward AI also vary widely, shaping levels of trust, acceptance, and willingness to integrate AI tools into clinical workflows [9]. Additionally, AI-related practice behaviors are influenced by concerns about professional autonomy, ethical accountability, and the need for human oversight, particularly in high-acuity settings where clinical judgment is essential [11]. Notably, none of the major international reviews—including Abdelmohsen and Al-Jabri [13], Bates et al. [5], or Ruksakulpiwat et al. [8]—explicitly examine how nurses’ AI competencies relate to patient safety culture. 

A further methodological challenge in the broader literature is that many studies examining AI-related perceptions draw on multidisciplinary samples—including physicians, allied health professionals, administrative staff, and students—without reporting nurse-specific quantitative data separately. To ensure valid conclusions about nurses’ AI readiness, nurse-specific quantitative data must be explicitly extractable rather than inferred from aggregated multidisciplinary results. When such disaggregation is absent, aggregated findings may not accurately represent nurses’ unique perspectives, training needs, or readiness for AI adoption. 

Accordingly, a refined understanding of nurses’ AI-related KAP requires careful attention to whether nurse-specific data are available and analytically separable from broader multidisciplinary samples. This issue is particularly relevant in critical care settings, where nurses’ decisions are closely linked to patient survival and where AI-supported care requires both technical competence and a strong safety culture. Therefore, this scoping review aimed to map current evidence, clarify conceptual and methodological gaps, and determine the extent to which existing studies provide nurse-specific insights into AI-related KAP and their potential relationship with patient-safety culture.

### Aim and Primary Research Question

The aim of this scoping review was to systematically map and synthesize existing international evidence on nurses’, nursing students’, and other included participants’ KAP related to AI, while examining the extent to which extractable nurse-specific data inform emerging evidence on patient safety culture. Guided by this aim, this review addressed the primary research question: “*What is known from the existing literature about the relationship between nurses’ KAP regarding AI and patient safety culture, based on studies in which nurse-specific quantitative data are explicitly extractable?*”.

## 2. Materials and Methods

This scoping review was conducted following the methodological framework proposed by Arksey and O’Malley [14], which outlines systematic stages for identifying, selecting, and synthesizing evidence in emerging fields. Their approach was further refined by Levac et al. [15], who emphasized iterative team engagement, enhanced analytical rigor, and clearer guidance for interpreting findings. To ensure transparency and methodological robustness, the review adhered to the Preferred Reporting Items for Systematic Reviews and Meta-Analyses Extension for Scoping Reviews (PRISMA-ScR) checklist, which provides structured reporting standards for scoping reviews and supports reproducibility in evidence synthesis [16]. This design was selected to comprehensively map the breadth of research examining nurses’, nursing students’, and other healthcare participants’ knowledge, attitudes, perceptions, and readiness regarding AI. Consistent with scoping review methodology, the review aimed to map existing evidence rather than evaluate intervention effectiveness.

### 2.1. Search Strategy

A comprehensive and systematic search strategy was developed to identify relevant literature examining nurses’, nursing students’, and other healthcare participants’ knowledge, attitudes, perceptions, and readiness regarding AI. The search process followed established guidance for transparent and reproducible evidence retrieval in scoping reviews, as recommended by the Joanna Briggs Institute (JBI) [17] and the PRISMA-ScR reporting standards [16]. Five major electronic databases—PubMed, CINAHL, Scopus, Web of Science, and ScienceDirect—were searched to capture interdisciplinary research spanning nursing, medicine, informatics, and computer science. The search covered the period from January 2021 to March 2026, a date range that was not prospectively protocolized and is therefore acknowledged as a methodological limitation. Although this timeframe reflects the rapid expansion of AI applications in healthcare, relevant pre-2021 studies may also exist.

Search terms combined controlled vocabulary (e.g., MeSH) and free-text keywords related to *artificial intelligence*, *machine learning*, *nurses*, *healthcare workers*, *knowledge*, *attitudes*, *perceptions*, *readiness*, *patient safety*, *safety culture*, *patient safety culture*, and *ethical concerns*. Boolean operators (AND/OR), truncation, and phrase searching were used to optimize sensitivity and specificity. Full executed search strings, exact search dates, platforms used, date and language limits, and the number of records retrieved from each database are documented to ensure transparency and reproducibility. Search strategies were adapted for each database to account for indexing differences. 

To ensure completeness, the reference lists of included studies and relevant systematic reviews were hand-searched, consistent with best practices for scoping review methodology [17]. A total of 18 records were identified through hand-searching, 7 of which were already captured through the five electronic databases and therefore did not increase the number of records imported into Covidence. All search activities were documented to support transparency and reproducibility (see Supplementary File S1).

### 2.2. Eligibility Criteria

Eligibility criteria were developed in accordance with established scoping review guidance to ensure clarity, transparency, and methodological rigor. Consistent with the JBI recommendations, inclusion and exclusion criteria were defined a priori to support systematic and reproducible study selection [17]. Studies were eligible for inclusion if they: (a) focused on nurses, nursing students, nursing faculty, or other healthcare professionals when nurse-specific quantitative data were explicitly extractable; (b) examined knowledge, attitudes, perceptions, readiness, or ethical concerns related to AI; (c) employed quantitative, qualitative, or mixed-methods designs; (d) were published in peer-reviewed journals; and (e) were available in English language. These criteria ensured that the review captured empirical evidence directly relevant to human experiences with AI in healthcare settings. In addition, systematic reviews were eligible when they provided synthesized evidence relevant to AI-related knowledge, attitudes, practice, or patient safety culture.

Exclusion criteria were applied to maintain conceptual focus and methodological quality. Studies were excluded if they centered solely on AI algorithm development without human participants, lacked primary data unless they were systematic reviews contributing synthesized evidence relevant to the review objectives, or were published as editorials, commentaries, conference abstracts, or non-peer-reviewed sources. Non-English publications were excluded due to feasibility constraints, consistent with common scoping review practices [16]. This language restriction may introduce bias and is acknowledged in the limitations of the review. Studies unrelated to healthcare or nursing contexts were also excluded. These criteria collectively ensured that the final evidence base reflected robust, relevant, and contextually meaningful research on AI perceptions among healthcare professionals. 

Because systematic reviews were eligible under the revised criteria, one systematic review was included in the final synthesis. To avoid double counting, all primary studies within the systematic review were cross-checked against the studies identified through our database search, confirming that no overlap existed with the empirical studies included in this scoping review.

### 2.3. Study Selection

The study selection process followed a structured and transparent approach consistent with PRISMA-ScR guidelines [16]. All records retrieved from database searches were imported into Covidence, an online systematic review management platform that supported automatic deduplication, blinded screening, conflict resolution, and documentation of reviewer decisions. The initial search yielded 4256 records, and Covidence identified and removed 657 duplicates, leaving 3599 records for title and abstract screening. Two reviewers independently screened all titles and abstracts using the predefined eligibility criteria. Any disagreements at this stage were resolved through consultation with a third reviewer, ensuring consistency and minimizing selection bias.

**Figure 1:**
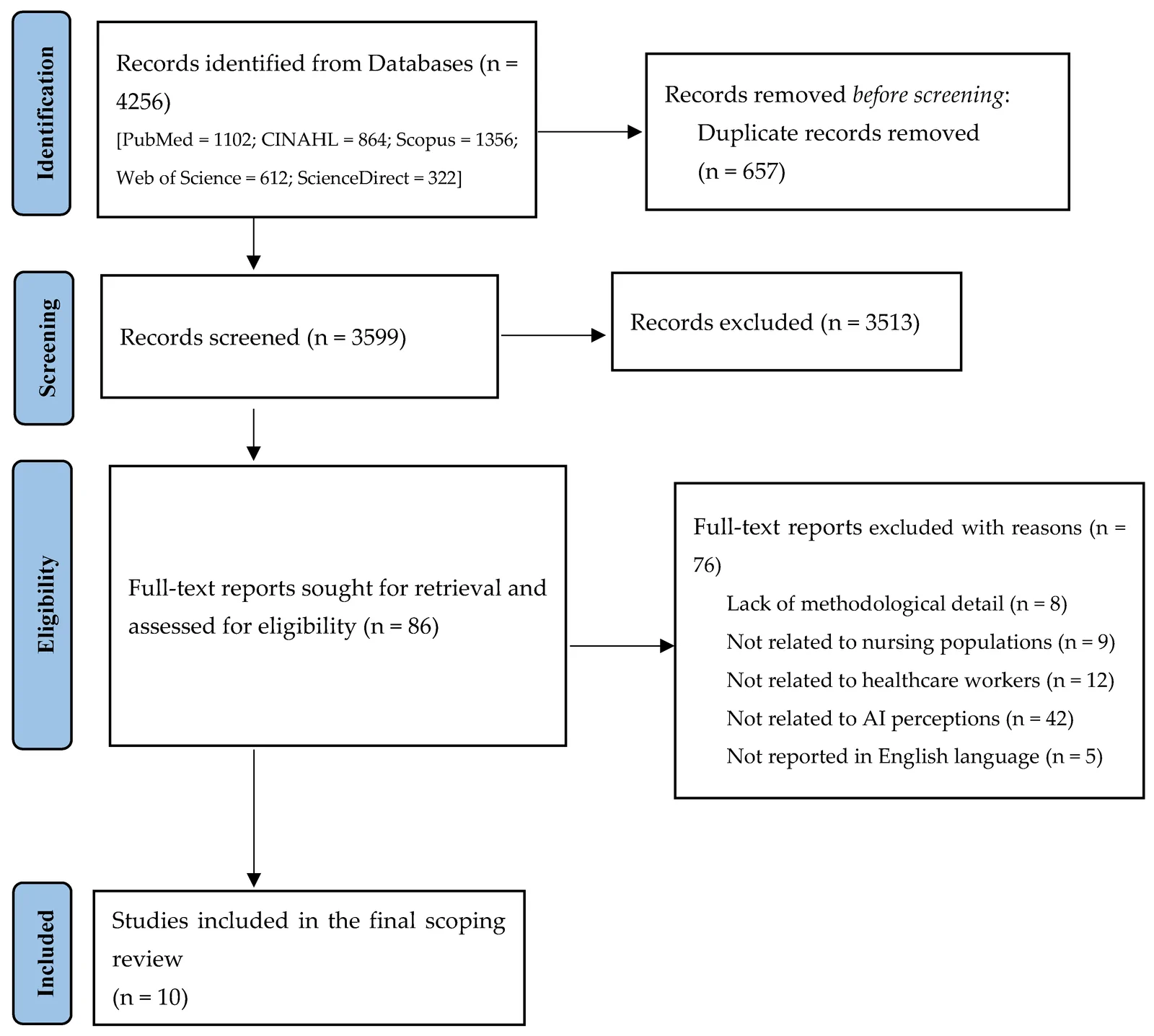
PRISMA 2020 Flow Diagram for this Scoping Review.

During title and abstract screening, 3513 records were excluded for not meeting inclusion criteria. The remaining 86 articles were retrieved and sought for full-text review and were assessed for eligibility. Full-text screening was also conducted in Covidence, with exclusion reasons documented systematically. Again, two reviewers independently assessed each article, and disagreements were resolved by a third reviewer to maintain methodological rigor. A total of 76 full-text articles were excluded for the following reasons: irrelevance to AI perceptions (n = 42), non-healthcare populations (n = 12), non-nursing populations (n = 9), insufficient methodological detail (n = 8), and non-English language publications (n = 5). Ultimately, 10 studies met all inclusion criteria and were included in the final synthesis, consisting of nine primary empirical studies and one systematic review that met the eligibility criteria permitting secondary evidence syntheses. To prevent double counting, all primary studies within the included systematic review were cross-checked against the empirical studies identified through our database search, confirming that no overlap existed. In the PRISMA flow diagram (Figure 1), non-English studies were classified as excluded at the full-text stage rather than “not retrieved,” to align with PRISMA-ScR guidance and avoid overlapping exclusion categories.

### 2.4. Data Extraction and Appraisal

Data extraction followed a structured and systematic process consistent with best-practice recommendations for scoping reviews outlined by the JBI [17]. A standardized extraction form was developed to ensure consistency across studies. For each included article, the following information was extracted: author(s), year of publication, and country, providing contextual and geographical insight into the evidence base; the objective of the study; and the study design, categorized according to JBI methodological classifications (see Table 1).

Detailed information on variables investigated was recorded, along with the specific tools or instruments used to gather data, such as validated questionnaires, Likert-scale surveys, or researcher-developed instruments. Data on participants and actual sample size were extracted to assess representativeness and population characteristics. Key findings were documented with emphasis on outcomes related to AI knowledge, attitudes, practice, and patient safety culture, aligning with the core aims of the review. Reported study limitations were captured to support critical appraisal and contextual interpretation. 

Each study also underwent methodological appraisal using the appropriate JBI critical-appraisal checklist based on its design (i.e., the JBI Checklist for Analytical Cross-Sectional Studies for the nine empirical studies and the JBI Checklist for Systematic Reviews and Research Syntheses for the included review) [16,17]. Appraisal items were recorded at the criterion level (“Yes,” “No,” “Unclear,” or “Not applicable”), and no numerical scores or global quality labels were assigned, in accordance with JBI guidance. For each study, an overall appraisal decision (“Include,” “Exclude,” or “Seek further information”) was documented. All ten studies were ultimately classified as “Include,” justified on the basis that most criteria were met, the review question was clearly stated, inclusion criteria were appropriate, search strategies were adequate, critical appraisal was conducted, synthesis methods were appropriate, and—where applicable—publication bias was not assessed but did not threaten inclusion. These justifications are summarized in the last column of Table 1. 

Consistent with scoping review methodology, these quality assessments were used descriptively and did not determine study inclusion or weighting in the synthesis. All extracted data and appraisal decisions were independently verified by two reviewers to ensure accuracy, transparency, and methodological rigor, with disagreements resolved through discussion or consultation with a third reviewer.

### 2.5. Data Analysis and Synthesis

Data analysis followed established methodological guidance for scoping reviews and narrative synthesis. Extracted data were first organized into descriptive numerical summaries, including study characteristics, populations, variables measured, and methodological features, consistent with the foundational framework of Arksey and O’Malley [14]. Narrative synthesis was then conducted following the structured guidance of Popay et al. [28], which emphasizes developing an initial synthesis, exploring relationships within and between studies, and assessing the robustness of the synthesis. This process involved iterative coding, categorization, and thematic mapping to identify patterns related to AI knowledge, attitudes, practice, and patient safety culture.

Analytical rigor was strengthened through team-based interpretation and reflexive discussion, aligning with recommendations by Levac et al. [15] for enhancing transparency and methodological clarity. The synthesis approach also adhered to the JBI Manual for Evidence Synthesis, which provides structured guidance for organizing and interpreting findings in scoping reviews [17]. Themes were compared across study designs and geographical contexts to identify convergences, divergences, and evidence gaps, with particular attention to AI literacy, educational preparedness, organizational readiness, ethical concerns, implementation barriers, and safety implications.

## 3. Results

### 3.1. Characteristics of the Included Studies

A total of 10 studies met the inclusion criteria, consisting of nine primary empirical cross-sectional studies and one systematic review. These studies were published between 2022 and 2026 and were conducted across diverse healthcare settings in China [19], Bangladesh [18], Pakistan [24], the Kingdom of Saudi Arabia (KSA) [20,21,22,23], Turkey [26], and the United Arab Emirates (UAE) [27]. The systematic review synthesized evidence from studies conducted in Egypt, the United States of America (USA), China, and Germany [25], offering a broader international perspective on nurses’, nursing students’, and other healthcare participants’ AI-related knowledge and attitudes.

Sample sizes in the original studies ranged from 95 to 1243 participants, while the systematic review summarized findings from five cross-sectional studies involving 1213 nurses. Participants included nurses, nursing students, nursing faculty, healthcare workers, and AI-related professionals, reflecting a wide range of clinical and educational contexts. Most studies used self-administered online questionnaires, frequently adapted from prior literature or validated through pilot testing and expert review. All primary empirical studies employed descriptive cross-sectional designs, with several incorporating inferential analyses such as logistic regression, chi-square tests, and correlation analyses.

### 3.2. Geographic Distribution

The 10 included studies represented a broad geographic spread across Asia and the Middle East. One study was conducted in China, examining AI knowledge and attitudes among nursing students, nurses, and other professionals [19]. Another study from Bangladesh assessed AI-related knowledge and attitudes among healthcare workers, most of whom were nurses [18]. Four studies were carried out in the KSA, focusing on nurses, nursing faculty, students, and mixed healthcare professionals [20].

In addition, one study from Pakistan explored AI perceptions among healthcare students and professionals [24]. A study from the UAE examined organizational readiness for AI integration among registered nurses [27]. Another study from Turkey investigated operating room nurses’ knowledge and ethical concerns regarding AI [26]. The systematic review incorporated studies conducted in Egypt, the USA, China, and Germany, thereby extending the evidence base beyond the Asian and Middle Eastern contexts [25].

### 3.3. Measurement Approaches

Across the 10 included studies, researchers primarily relied on structured self-administered questionnaires to assess nurses’, nursing students’, and other participants’ knowledge, attitudes, perceptions, and readiness related to AI. These instruments commonly incorporated Likert-type scales, enabling the measurement of agreement levels, perceived competence, ethical concerns, and willingness to adopt AI in clinical practice. Several studies adapted previously validated tools or refined their questionnaires through pilot testing and expert review to ensure clarity and content validity [18,23]. The questionnaires typically included sections assessing knowledge of AI concepts, attitudes toward AI integration, perceived benefits and risks, and AI-related practices, with some studies expanding their scope to evaluate organizational readiness [27] and patient safety culture [21]. The systematic review employed the AXIS tool, a 20-item critical appraisal instrument specifically developed to assess the methodological quality, reporting standards, and risk of bias in cross-sectional studies [29].

### 3.4. Study Focus and Patterns Across Studies

Across the included studies, the primary focus was on assessing knowledge, attitudes, perceptions, and readiness related to AI in healthcare and nursing practice. Several studies also examined ethical concerns, including issues related to privacy, job displacement, and the potential erosion of empathy in patient care [19,21,26]. Other studies explored AI practices and organizational readiness, particularly in the KSA [22] and the UAE [27]. All included studies were conducted in LMIC or middle-income countries (Bangladesh, Pakistan, China, KSA, Turkey, UAE). No high-income country data were included except indirectly through the systematic review [25]. One study specifically investigated the relationship between KAP of AI and patient safety culture, but did not establish causal or generalizable associations between these variables due to its cross-sectional design [21]. Another study identified positive associations among AI-related KAP domains [22], while a separate study reported a positive correlation between knowledge and attitudes toward AI adoption [18]. Two studies did not examine relationships among KAP variables; however, they reported significant differences in AI-related knowledge and attitudes across healthcare professional groups [19] and according to nurses’ sociodemographic characteristics [20]. Demographic-associated factors such as age, education level, and prior exposure to AI were also examined, with several studies identifying these factors as having significant associations with AI-related knowledge and attitudes [23,24,25]. The remaining two studies did not establish associations among AI-related KAP variables or patient safety culture [26,27].

Overall, AI knowledge levels varied across studies, ranging from low [19,25,27] to good or satisfactory [18,20,21,22,23,24,26], depending on context and population, despite widespread exposure to AI technologies in clinical and educational settings [18,19]. In contrast, attitudes toward AI were predominantly positive, with participants frequently expressing optimism about AI’s potential to enhance workflow efficiency, diagnostic accuracy, and overall care quality [20,22]. At the same time, ethical concerns regarding the integration of AI into healthcare practice were commonly reported, particularly regarding data privacy, job displacement, and the potential reduction of human empathy in patient care [21,26]. Demographic factors such as younger age, higher educational attainment, and prior exposure to AI were consistently associated with higher knowledge scores and more favorable attitudes [23,24].

Across multiple settings, nurses emphasized the need for structured AI training and formal curricular integration, highlighting a perceived gap between emerging technological demands and current educational preparation [22,27]. Importantly, only one study identified limited evidence of a connection between AI knowledge, attitudes, and actual AI-related practices and patient safety culture. In the KSA, Eldesoky et al. [21] found that although healthcare providers demonstrated moderate levels of AI-related knowledge and attitudes, these factors were significantly associated with patient safety culture scores. This finding suggests that AI-related perceptions have not yet become sufficiently embedded in safety-related behaviors or organizational systems to be associated with patient safety outcomes.

### 3.5. Narrative Synthesis

The ten included studies collectively provide a broad examination of nurses’, nursing students’ and other included healthcare participants’ KAP regarding AI across diverse clinical and educational settings. Although conducted in different countries and among varied professional groups, the studies revealed several converging themes that illuminate the current state of AI awareness and integration in healthcare.

Across settings, AI knowledge levels varied across studies, ranging from low to satisfactory depending on context and participant group, even among participants who reported routine exposure to AI-enabled technologies in their work or academic environments [18,19]. Participants often demonstrated familiarity with general AI concepts but lacked deeper understanding of AI applications, limitations, and implications for clinical decision-making. This pattern was consistent across nurses, students, faculty, and mixed healthcare worker groups.

Despite modest knowledge levels, attitudes toward AI were predominantly positive. Participants across multiple countries expressed optimism about AI’s potential to enhance workflow efficiency, improve diagnostic accuracy, and support clinical decision-making [20,22]. Positive attitudes were particularly evident among younger professionals and those with prior exposure to AI technologies, suggesting a growing openness to AI integration within the healthcare workforce.

Alongside positive attitudes, ethical concerns emerged as a prominent theme. Participants frequently expressed apprehension regarding data privacy, job displacement, algorithmic transparency, and the potential erosion of empathy in patient care. These concerns were especially pronounced among operating room nurses in Turkey and healthcare providers in the KSA [21,26]. Such concerns highlight the tension between enthusiasm for AI’s benefits and uncertainty about its broader implications for professional identity and patient relationships.

Several studies identified demographic factors associated with AI-related perceptions. Younger age, higher educational attainment, and prior exposure to AI were consistently associated with higher knowledge levels and more favorable attitudes [23,24]. These findings suggest that generational familiarity with digital technologies and educational opportunities may be associated with readiness for AI adoption.

Across regions, nurses consistently emphasized the need for structured AI training and formal curricular integration. Participants expressed a desire for competency-based training, hands-on exposure to AI tools, and clearer institutional guidance to support safe and effective AI use in practice [22,27]. The widespread call for training underscores a global gap between emerging technological demands and current educational preparation.

Of the 10 included studies, only one directly examined the relationship between AI-related knowledge, attitudes, and patient safety culture [21]. In this study, healthcare providers demonstrated moderate AI knowledge and moderately positive attitudes. Patient safety culture was also rated as moderate. Multiple linear regression analysis showed that both AI knowledge and AI attitude were significant positive predictors of patient safety culture. The model explained 60% of the variance, indicating a substantial statistical association, and the overall model fit was confirmed by a significant F-test. Attitude toward AI showed a stronger statistical association with knowledge, suggesting that perceptions and openness to AI may play a more critical role than technical familiarity. However, because this is the only study examining this relationship, the evidence cannot be considered conclusive or generalizable despite the statistically significant findings.

Overall, the evidence indicates that nurses, nursing students, and other included healthcare participants are generally receptive to AI integration but lack the knowledge, training, and structural support necessary for effective and safe implementation. The convergence of positive attitudes, ethical concerns, demographic-associated factors, and training needs underscores the importance of developing comprehensive educational strategies, organizational policies, and regulatory frameworks to guide AI adoption in healthcare. The limited and inconclusive evidence regarding any relationship between AI perceptions and patient safety culture reflects the fact that only one study examined this relationship, and its findings remain insufficient to establish broader conclusions. This suggests that AI-related perceptions have not yet been sufficiently integrated into safety-related behaviors or organizational systems.

## 4. Discussion

The findings of this review align closely with a growing body of international evidence demonstrating that nurses, nursing students, and other included healthcare participants worldwide possess variable levels of AI knowledge, despite increasing exposure to AI-enabled technologies. Similar to the patterns observed across the 10 included studies (nine empirical studies and one systematic review), research from the KSA found that critical care nurses demonstrated only moderate AI knowledge, with many reporting uncertainty about AI’s technical capabilities [30]. In Germany, Sommer et al. [31] reported that only one quarter of nurses considered themselves knowledgeable about AI, even though most acknowledged its growing relevance in healthcare. Comparable results have been reported in China, where nursing students demonstrated limited AI literacy but expressed strong motivation to learn more about AI [32], and in India, healthcare professionals demonstrated limited awareness and inadequate training related to digital health technologies, including AI-enabled systems, yet many still expressed generally positive attitudes toward adopting such innovations in practice [33]. Studies from Canada [34] and the USA [35] similarly highlight that nurses show interest in AI-enabled clinical support but often lack sufficient technical understanding, a pattern echoed in the USA, where perioperative nurses expressed enthusiasm for AI-assisted handoffs yet reported notable knowledge gaps and usability concerns [35]. Collectively, these findings reinforce the interpretation that AI literacy remains underdeveloped globally, even as enthusiasm for AI’s potential benefits continues to grow.

The ethical concerns identified in this review—particularly those related to privacy, job displacement, algorithmic transparency, and the erosion of empathy—are strongly echoed in international research. In Australia, nurses expressed apprehension that AI could undermine the relational aspects of care and diminish professional autonomy [36]. Similar concerns were reported in the United Kingdom (UK), where healthcare professionals expressed worries that AI could undermine essential elements of clinical care, including empathy, data security, and the reliability of decision-making processes [37]. German nurses also voiced concerns about losing control over clinical processes and the potential dehumanization of care [31]. In Brazil, nurses worried that AI could replace certain nursing tasks, altering the nature of the profession [38]. These international findings mirror the ethical tensions observed in the included studies, particularly among nurses in high-acuity environments such as operating rooms. The convergence of these concerns across countries underscores the need for robust ethical frameworks, transparent AI governance, and professional guidelines to support nurses as AI becomes more deeply integrated into healthcare systems.

The demographic patterns observed in this review, especially the associations among younger age, higher education, and prior exposure to AI, are consistent with findings from multiple international studies. In Korean healthcare students, higher AI literacy was significantly associated with more positive attitudes toward AI and a stronger intention to use AI in clinical contexts, while no significant differences were observed by gender, major, or year of study [39]. Similar associations were observed in China, where nursing students with stronger digital literacy demonstrated more positive perceptions of AI [32]. In the KSA, Alrashedi et al. [30] found that younger and more highly educated nurses were more receptive to AI integration, while older nurses expressed greater apprehension. German nurses with prior AI exposure were also more likely to perceive AI positively and express readiness for its integration into clinical workflows [31]. These demographic patterns suggest that generational familiarity with digital technologies and educational opportunities may be associated with greater readiness for AI adoption. These consistent demographic patterns across countries highlight the importance of targeted training strategies that address the needs of older or less technologically experienced professionals.

The strong demand for structured AI training identified in this review is mirrored in international studies, which consistently highlight the inadequacy of current educational preparation for AI integration. In Canada, nurses reported limited opportunities for AI-related training and emphasized the need for formal education that includes both theoretical and practical components [34]. Brazilian nurses similarly identified significant training gaps and expressed a desire for competency-based AI education [38]. In China, nursing students reported limited exposure to AI-related content in their curricula and emphasized the need for more comprehensive and structured AI training [32]. Studies from Australia [36] and the UK [37] also highlighted the need for structured AI training to support safe and effective implementation. These international findings reinforce the conclusion that AI education remains underdeveloped globally, and that healthcare systems must prioritize the integration of AI competencies into pre-licensure programs, continuing professional development, and organizational training frameworks.

This review identified only one study that examined the relationship between AI-related knowledge, attitudes, and patient safety culture. Eldesoky et al. [21] found that both knowledge and attitudes toward AI were positively associated with patient safety culture, with attitudes showing a stronger statistical association. These findings indicate a measurable association between AI readiness and patient safety culture; however, because this evidence is derived from a single cross-sectional study conducted in one hospital, it remains insufficient to establish consistency, causality, or generalizability across nursing populations or clinical environments. International evidence on this topic remains sparse. In Norway, Sockolow et al. [40] found no strong relationship between nurses’ AI competence and their perceptions of patient safety culture, suggesting that AI awareness has not yet translated into safety-related behaviors or organizational norms. Similarly, Alrashedi et al. [30] reported that while Saudi nurses expressed positive attitudes toward AI, these attitudes did not correspond to measurable differences in safety practices. German nurses also viewed AI as beneficial but did not perceive it as directly connected to patient safety outcomes [31]. These studies highlight that AI integration remains too early in many healthcare systems for consistent patterns to emerge, and the global evidence base remains extremely limited. Furthermore, because many international studies use multidisciplinary samples without reporting nurse-specific quantitative data separately, the ability to draw conclusions about nurses’ unique contributions to patient safety culture is methodologically constrained. The inclusion of one systematic review in this scoping review further underscores the scarcity of robust empirical evidence, as the systematic review itself did not identify consistent associations between AI competencies and patient safety culture across countries. This highlights the need for future research exploring how AI competencies, organizational readiness, workflow integration, and safety culture may interact in ways that are associated with how clinical practice is shaped.

Overall, the evidence suggests that while nurses are increasingly open to AI integration, their knowledge and practical readiness remain limited. Ethical concerns, demographic-associated factors, and substantial training gaps consistently inform perceptions across diverse settings. Although one study demonstrated a positive association between AI knowledge, attitudes, and patient safety culture, the overall evidence base is too limited to conclude that AI readiness is consistently associated with safety culture in a generalizable way. Moreover, the study examining patient safety culture was conducted in a multidisciplinary healthcare sample and did not report nurse-specific findings separately; therefore, the observed association cannot be attributed exclusively to nurses. Future research must prioritize nurse-specific quantitative data, rigorous methodological designs, and explicit examination of how AI-related competencies intersect with patient safety culture. Strengthening AI literacy, embedding structured training into professional education, and addressing ethical and organizational challenges will be essential to support safe and effective AI adoption.

### 4.1. Strengths and Limitations of the Scoping Review

This scoping review offers several strengths that enhance the credibility and relevance of its findings. The review synthesizes evidence from multiple countries across Asia, the Middle East, Europe, and the Americas, providing a broad international perspective on nurses’, nursing students’ and other included healthcare participants’ knowledge, attitudes, and readiness regarding AI. The inclusion of both primary empirical studies and one systematic review—consistent with the eligibility criteria—strengthens the comprehensiveness of the evidence base and allows for the integration of synthesized cross-country insights. The use of a structured methodological approach, guided by established scoping review frameworks, further supports the rigor and transparency of the review process. Additionally, the integration of recent international studies allows for meaningful cross-context comparisons, highlighting global patterns and shared challenges in AI adoption.

However, several limitations should be acknowledged. The evidence base is dominated by cross-sectional studies, which restricts the ability to infer causal relationships or examine changes in AI perceptions over time. Variability in measurement tools, sample characteristics, and operational definitions of AI constructs may limit comparability across studies. Most included studies relied on self-reported data, which may introduce response bias. Furthermore, the geographic distribution of studies remains uneven, with limited representation from Africa, South America, and high-income Western countries. Although one systematic review was included, its synthesized findings were also constrained by the limited number and methodological diversity of available primary studies. These gaps underscore the need for more diverse, longitudinal, and methodologically robust research to advance understanding of AI integration in healthcare.

### 4.2. Implications of the Review Findings

The implications of this review highlight several important considerations for healthcare policy, nursing practice, and future research. The findings indicate a growing need for healthcare policies that proactively support the safe and equitable integration of AI across health systems. Policymakers should prioritize the development of national AI competency frameworks, standardized guidelines for ethical AI use, and regulatory mechanisms that ensure transparency, accountability, and data protection. Given the variable but often limited levels of AI literacy observed across studies, policies must also mandate investment in workforce development, including funding for AI training programs and incentives for institutions to adopt evidence-based digital transformation strategies. Strengthening governance structures and establishing clear accountability pathways will be essential to mitigate risks related to algorithmic bias, privacy breaches, and workforce displacement.

For nursing practice, the results underscore the need to embed AI competencies into routine clinical workflows and professional standards. Nurses require structured opportunities to develop foundational AI skills, including understanding AI outputs, recognizing limitations, and applying clinical judgment when interacting with AI-enabled tools. Clinical leaders should foster environments that encourage responsible AI use, promote interdisciplinary collaboration with data scientists and informaticians, and ensure that AI systems complement rather than replace the relational and humanistic dimensions of nursing care. As AI becomes more prevalent, nurses will play a critical role in advocating for patient safety, ethical implementation, and equitable access to AI-supported services.

Future efforts should focus on strengthening the evidence base for AI integration in healthcare by supporting longitudinal, multi-site, and mixed-methods studies that examine how AI competencies evolve over time and are associated with clinical outcomes. There is a particular need for research that reports nurse-specific quantitative data, given that many existing studies rely on multidisciplinary samples that do not allow for disaggregated analysis. Studies should also explicitly examine how AI-related KAP intersect with patient-safety culture, as only one included study explored this relationship and its findings were inconclusive. Research should also investigate the effectiveness of AI training interventions, the ways in which AI may be associated with professional identity and workflow redesign, and the organizational conditions linked to successful adoption. Expanding research to underrepresented regions—particularly Africa, South America, and low-resource settings—will be essential to ensure that global AI strategies are inclusive, context-appropriate, and responsive to diverse healthcare needs.

## 5. Conclusions

This scoping review provides a comprehensive synthesis of global evidence on nurses’, nursing students’, and other included healthcare participants’ knowledge, attitudes, perceptions, and readiness regarding AI. Across diverse international contexts, the findings reveal a recurring but context-dependent pattern: although nurses generally express positive attitudes toward AI and acknowledge its potential to enhance efficiency, diagnostic accuracy, and clinical decision-making, their knowledge and practical readiness remain limited. This persistent gap between enthusiasm and competency underscores the early stage of AI integration in healthcare and highlights the need for structured, accessible, and context-appropriate educational initiatives. Ethical concerns—including data privacy, algorithmic transparency, job displacement, and the potential erosion of empathy—emerged as central themes. These concerns reflect broader tensions between technological innovation and the preservation of humanistic, patient-centered care. Addressing these issues will require clear ethical guidelines, transparent governance structures, and organizational cultures that prioritize accountability and patient rights.

A critical finding of this review is the limited evidence regarding the relationship between AI knowledge, attitudes, and patient safety culture. Only one included empirical study examined this relationship, and it reported a positive association, suggesting that greater AI knowledge and more favorable attitudes may be associated with stronger perceptions of patient safety culture. However, this evidence is based on a single cross-sectional study conducted in one setting and cannot be generalized or used to infer causality. The inclusion of one systematic review further highlights the scarcity of robust empirical evidence, as the synthesized findings also indicated limited and inconsistent associations between AI competencies and patient safety culture across countries. Moreover, because many existing studies rely on multidisciplinary samples without reporting nurse-specific quantitative data separately, the ability to draw valid conclusions about nurses’ unique contributions to patient safety culture is substantially limited. Importantly, the only study examining the relationship between AI-related factors and patient safety culture was conducted in a multidisciplinary healthcare sample and did not report nurse-specific results separately. Therefore, its findings should not be interpreted as evidence specific to nurses. Overall, the findings indicate that available studies suggest generally favorable attitudes toward AI alongside variable knowledge, limited formal training, and persistent ethical and organizational concerns. For nurses specifically, the available evidence supports conclusions regarding AI-related attitudes, knowledge, training needs, and implementation concerns; however, evidence linking these factors to patient safety culture cannot currently be attributed exclusively to nurses. Evidence linking these factors to patient safety culture remains extremely limited, with only one eligible cross-sectional study identified. Further longitudinal and multisite research is required before the nature or direction of this relationship can be established.

## Figures and Tables

**Table 1:** Characteristics of the Included Studies.

Author/s, Year, Country	Objective of the Study	Study Design	Variables and Tools Used	Participants, Sample and Population-Specific Data	Key Findings	Limitations	JBI Appraisal
Rony et al. [18] Bangladesh	To investigate healthcare workers’ knowledge and attitudes regarding AI adoption in healthcare in Dhaka	Cross-sectional survey	Variables: AI knowledge (14 MCQs), attitudes toward AI (Likert scale), demographics. Tools: Literature-based questionnaire; content validity; Cronbach’s α overall = 0.85 (knowledge α = 0.82; attitude α = 0.88); exploratory factor analysis.	Healthcare workers (physicians, nurses, technicians, admin staff); n = 431. Multidisciplinary; nurse-specific data not separated.	Younger HCWs (18–35), physicians, hospital workers, and full-time staff had higher odds of good knowledge and positive attitudes. Attendance at AI conferences and learning via research articles/journals associated with better knowledge and attitudes. Strong positive correlation between knowledge and positive attitudes (r = 0.89) and between poor knowledge and negative attitudes (r = 0.65).	Single-city sample; mix of random and convenience sampling; self-report; cross-sectional (no causality); limited to Dhaka and to those meeting education/experience criteria.	Include This study met most JBI criteria, including clear aims, validated tools, and appropriate analytical methods. Confounding was not fully addressed.
Wang et al. [19] China	To explore knowledge, attitudes, concerns, and ethical views toward AI in nursing among various professional categories in China	Online cross-sectional survey	Variables: Knowledge of AI and AI in nursing (3 items, 4-point Likert); attitudes toward AI in nursing (9 items, 4-point Likert); concerns and ethical issues (multiple-choice); demographics. Tools: Researcher-developed questionnaire; Cronbach’s α for attitude = 0.860; KMO = 0.768; Bartlett *p* < 0.001; EFA (2 factors, 65.79% variance).	Nursing students, nurses, other healthcare professionals, AI-related professionals, and others; n = 1243. Multidisciplinary; nurse-specific data not separated	57% knew only “a little” about AI; 4.7% knew nothing. For AI in nursing, 64.7% knew only “a little” and 13.4% knew nothing. Despite low knowledge, >50% agreed/strongly agreed with all positive attitude items—overall positive attitudes. All participants expressed concerns; 95.7% felt medical ethics around AI in nursing must be strengthened.	Snowball sampling; online, self-report; cross-sectional; knowledge section brief; reliability not tested for knowledge/concern items; China-only sample.	Include The study demonstrated clear objectives, validated scales, and appropriate statistical analysis. Confounding was not addressed.
Alruwaili et al. [20] KSA	To assess nurses’ awareness and attitudes toward AI-integrated tools in clinical practice and explore demographic associations.	Descriptive cross-sectional survey	Variables: AI awareness (7 yes/no items), attitudes toward AI (20-item GAAIS), demographics. Tools: Nurses’ AI Knowledge Questionnaire (Swed et al.); GAAIS (Schepman & Rodway); Cronbach’s α = 0.795 (knowledge), 0.88 (positive GAAIS), 0.82 (negative GAAIS).	Registered nurses in three governmental hospitals (Jouf region); n = 220. Nurse-only sample	70.9% had basic information about AI; 67.3% had heard of AI in medicine; 58.2% had used some AI means; 60% knew about ML. Overall attitudes were moderately positive (M ≈ 3.5/5) but with notable concerns about control, errors, surveillance, and ethics. Younger nurses and those with undergraduate degrees showed more positive attitudes; female nurses had more negative attitudes than males.	Convenience sampling; three hospitals in one region; self-report; cross-sectional; no objective measure of AI competence; limited generalizability beyond governmental hospitals.	Include Most criteria were met, including validated instruments and appropriate analysis. Confounders were partially addressed.
Eldesoky et al. [21] KSA	To examine healthcare providers’ knowledge and attitudes toward AI and their relationship with patient safety culture.	Descriptive correlational cross-sectional study	Variables: AI knowledge (20 MCQs), AI attitude (20-item attitude scale), patient safety culture (HSOPSC, 42 items), demographics. Tools: Structured Knowledge and Attitude Questionnaire on AI (researcher-developed; α = 0.91); HSOPSC (AHRQ; α = 0.88).	Physicians, nurses, and technicians at Najran University Hospital; n = 238. Multidisciplinary; nurse-specific data not separated	The findings demonstrated that healthcare providers’ knowledge and attitude toward AI were significant predictors of patient safety culture, jointly explaining 60% of its variance (R^2^ = 0.60). Knowledge (B = 0.296, *p* = 0.000) and attitude (B = 0.502, *p* = 0.000) both showed positive associations with safety culture, with attitude exerting the stronger association (Beta = 0.496 vs. 0.325). The significant F-test (F = 170.914, *p* = 0.000) confirmed that the regression model provided a robust fit for predicting patient safety culture.	Single university hospital; convenience sampling; self-report; cross-sectional (no causal inference); AI knowledge tool context-specific; no longitudinal follow-up.	Include This study met nearly all JBI criteria, including identification and management of confounders and strong analytical methods.
Mariano et al. [22] KSA	To assess knowledge, attitudes, practices (KAP), and their relationships regarding AI among nurses, nursing faculty, and students in Saudi Arabia.	Cross-sectional descriptive correlational survey	Variables: AI knowledge (10 MCQs), attitudes (12 Likert items), practices/experience (12 Likert items), demographics. Tools: Adapted from De Simone, Ergin, Perrier, Swed et al.; pilot-tested; Cronbach’s α: knowledge = 0.91, attitude = 0.88, practice = 0.79.	Nursing students, staff nurses, and nursing faculty from multiple universities, colleges, hospitals, and clinics; n = 349. Nurse-only sample, including nursing students	Mean knowledge score 7.37/10 (73.7% correct)—“satisfactory” knowledge. Attitudes mildly positive (overall mean ≈ 3.06/5); many agreed AI can reduce costs, improve diagnosis, and is essential in nursing, but a substantial proportion feared job replacement and ethical/data-security threats. Practices moderately positive: many reported some exposure and confidence, but formal training was limited; strong support for specific AI training and ethics education. Significant positive correlations among KAP.	Convenience and snowball sampling; online self-report; cross-sectional; over-representation of females and government sector; no objective practice measures.	Include The study met most criteria, with validated tools and appropriate analysis. Confounding was not addressed.
Serbaya et al. [23] KSA	To determine levels of AI-related KAP among healthcare workers in private polyclinics in Jeddah and explore associated factors.	Cross-sectional survey	Variables: AI awareness/knowledge (6 items), attitudes (8 items), behaviors/practice (3 items), demographics. Tools: Researcher-developed questionnaire; content validated; Cronbach’s α: knowledge = 0.79, attitude = 0.77, behavior = 0.62.	Physicians, nurses, administrative staff, and others in private polyclinics; n = 361. Multidisciplinary; nurse-specific data not separated	Median knowledge score 5/6 (IQR 3–6)—overall good awareness; 74.5% felt they understood basic computational principles; 70% agreed AI has useful medical applications. Median attitude score 5/8—generally positive; 83% thought AI useful in their work; 75% believed AI can reduce medical errors; 58% worried AI might replace them. Practice score median 0/3 (IQR 0)—indicating limited “good” behavior items as coded, but most agreed AI lacks flexibility, struggles with empathy, and has constraints. Nurses had highest knowledge scores; administrative staff had highest practice scores.	Convenience sampling; private sector only; behavior scale short and lower reliability; cross-sectional; self-report; limited detail on AI exposure types.	Include Clear aims, validated tools, and appropriate analysis were present. Confounding was partially addressed.
Habib et al. [24] Pakistan	To analyze knowledge, attitudes, and perceptions of healthcare students and professionals regarding AI use in healthcare in Pakistan.	Cross-sectional online survey	Variables: General AI understanding, AI in education, AI capabilities in healthcare and public health, impact on careers, social/ethical consequences; demographics. Tools: Researcher-developed questionnaire via Google Forms.	Doctors, nurses, allied health professionals, and students; n = 610. Multidisciplinary; nurse-specific data not separated	Most had no formal AI training, yet showed generally good conceptual knowledge and optimistic attitudes toward AI’s role in diagnosis, prognosis, capacity planning, and quality improvement. 66.4% felt AI training should start at undergraduate level; majority wanted 1–5 h/month of AI learning. 59.5% believed AI will substitute some healthcare jobs; many anticipated new ethical, social, and equity challenges. Higher education level associated with more positive attitudes and perceptions.	Online convenience sample; self-report; no validated scales reported; cross-sectional; Pakistan-only; potential selection bias toward tech-interested respondents.	Include The study met most criteria, including validated tools and appropriate descriptive analysis. Confounding was not addressed.
Sandanasamy et al. [25] multi-country in a systematic review	To systematically review nurses’ knowledge and attitudes toward AI and related factors.	Systematic review of cross-sectional studies	Variables (across included studies): AI knowledge, attitudes, and associated factors (education, gender, facility type). Tools: AXIS tool for quality appraisal; narrative synthesis of 5 cross-sectional studies (Egypt, USA, China, Germany).	Nurses across 5 included studies; total n = 1213. Nurse-only sample	Pooled average knowledge score ≈ 21.93/100—indicating poor AI knowledge among nurses. Average positive attitude ≈ 67.19/100—generally high positive attitudes. Education level, gender, and type of healthcare facility significantly associated with knowledge in at least one study; education level associated with attitude in one study. Concludes that policy and management should target education, gender gaps, and facility context to improve AI readiness.	Only English-language cross-sectional studies; small number of studies (n = 5); heterogeneity precluded meta-analysis; no gray literature; possible missed studies; relies on self-report data from primary studies.	Include Most criteria met; the review demonstrated a clear question, appropriate inclusion criteria, a transparent and comprehensive search strategy, and suitable critical appraisal and synthesis methods. Publication bias assessment was only partially addressed, but overall the AXIS-based appraisal and the review processes were appropriate and aligned with JBI requirements.
Durukan et al. [26] Turkey	To assess operating room nurses’ knowledge and opinions about AI.	Descriptive cross-sectional study	Variables: Knowledge about AI (sources, level), opinions/attitudes (benefits, ethical concerns, workload, quality of care), demographics. Tools: Google Forms questionnaire with sections on personal info, AI knowledge, and opinions; descriptive statistics.	Operating room nurses in a private hospital; n = 95. Nurse-only sample	Nurses’ knowledge about AI was reported as high; social media was the most common information source, though perceived as unreliable. Nurses expressed ethical concerns (lack of empathy, potential malfunctions) but believed AI could reduce workload and improve quality of care.	Single private hospital; small sample; descriptive only (no inferential analysis reported); self-report; cross-sectional; limited psychometric detail on the tool.	Include Clear aims, validated tools, and appropriate analysis were present. Confounding was not addressed.
Abuzaid et al. [27] UAE	To assess nurses’ knowledge, attitudes, willingness, and organizational readiness for integrating AI into nursing practice.	Exploratory cross-sectional electronic survey	Variables: AI knowledge (self-rated understanding, training, curriculum views), skills acquisition, perceptions/attitudes (role, threats, limitations, feelings), organizational readiness (strategy, leadership, integration), demographics. Tools: Adapted validated survey from prior AI studies; piloted with senior nurses and educators; online via Google Forms.	Registered nurses in UAE hospitals and clinics; n = 553. Nurse-only sample	30% said knowledge came only from media; 26% were familiar but not confident to apply; 12% very comfortable; 10% had no idea; 75% agreed nursing curriculum should include basic AI; 78% felt AI should be taught at undergraduate level; 83% at postgraduate level. Many were self-taught; formal training limited. Most agreed AI plays an important role and will be present in many nursing applications; 57% felt AI could threaten some nursing careers; many were excited but also aware of challenges. Organizational readiness was mixed, with many unsure about AI strategies or leadership.	Convenience sampling; UAE-only; self-report; cross-sectional; no detailed psychometrics reported; possible non-response and selection bias.	Include The study met most criteria, including validated tools and appropriate descriptive analysis. Confounding was not addressed.

## Data Availability

No new data were created or analyzed in this study.

## Supplementary Materials

The following supporting information can be downloaded at: https://www.mdpi.com/article/10.3390/nursrep16100370/s1, File S1: Complete Search Strategy and Results (January 2021–March 2026).

## Public Involvement Statement

There was no public involvement in any aspect of this research.

## Guidelines and Standards Statement

This manuscript was drafted against the PRISMA-ScR (the Preferred Reporting Items for Systematic Reviews and Meta-Analyses extension for Scoping Reviews [PRISMA-ScR]) guidelines [16].
