# Supplementary material for: Artificial Intelligence Readiness in Nursing: A Scoping Review of Knowledge, Attitudes, Practice, and Emerging Evidence on Patient Safety Culture
Source: Nurs Rep. 2026 Oct 19;16(10):370. doi: 10.3390/nursrep16100370 (PMC13708583; doi:10.3390/nursrep16100370)
Supplement: Supplementary file 1 [file nursrep-16-00370-s001.zip › nursrep-4348982-supplementary.pdf]

Supplementary File S1: Complete Search Strategy and Results (January 2021–March 2026)

Database	Platform	Search Date	Search String (Executed)	Limits Applied	Records Retrieved
PubMed	NCBI	15-Mar-26	(("artificial intelligence"[MeSH] OR "machine learning"[MeSH] OR "artificial intelligence"[tiab] OR "machine learning"[tiab] OR AI[tiab]) AND ("nurses"[MeSH] OR nursing[tiab] OR "nursing students"[tiab] OR "healthcare workers"[tiab]) AND (knowledge[tiab] OR attitudes[tiab] OR perceptions[tiab] OR readiness[tiab] OR "patient safety"[tiab] OR "safety culture"[tiab] OR "patient safety culture"[tiab] OR "ethical concerns"[tiab]))	English; 2021–2026	1,102
CINAHL	EBSCOhost	16-Mar-26	(MH "Artificial Intelligence" OR MH "Machine Learning" OR TI ("artificial intelligence" OR "machine learning" OR AI) OR AB ("artificial intelligence" OR "machine learning" OR AI)) AND (MH "Nurses" OR MH "Nursing Students" OR TI (nurs OR "healthcare workers") OR AB (nurs OR "healthcare workers")) AND (TI (knowledge OR attitudes OR perceptions OR readiness OR "patient safety" OR "safety culture" OR "patient safety culture" OR "ethical concerns") OR AB (knowledge OR attitudes OR perceptions OR readiness OR "patient safety" OR	English; 2021–2026	864

			"safety culture" OR "patient safety culture" OR "ethical concerns"))**		
Scopus	Elsevier	17-Mar-26	TITLE-ABS-KEY("artificial intelligence" OR "machine learning" OR AI) AND TITLE-ABS-KEY(nurs OR "nursing students" OR "healthcare workers") AND TITLE-ABS-KEY(knowledge OR attitudes OR perceptions OR readiness OR "patient safety" OR "safety culture" OR "patient safety culture" OR "ethical concerns")*	English; 2021–2026	1,356
Web of Science	Clarivate	17-Mar-26	TS=("artificial intelligence" OR "machine learning" OR AI) AND TS=(nurs OR "nursing students" OR "healthcare workers") AND TS=(knowledge OR attitudes OR perceptions OR readiness OR "patient safety" OR "safety culture" OR "patient safety culture" OR "ethical concerns")*	English; 2021–2026	612
ScienceDirect	Elsevier	18-Mar-26	("artificial intelligence" OR "machine learning" OR AI) AND (nurs OR "nursing students" OR "healthcare workers") AND (knowledge OR attitudes OR perceptions OR readiness OR "patient safety" OR "safety culture" OR "patient safety culture" OR "ethical concerns")*	English; 2021–2026	322

Hand-searching / Reference-list searching	Manual	March 18–20, 2026	Reference lists of included studies and relevant systematic reviews screened; additional records included if meeting eligibility criteria	No limits	Eighteen records were identified through hand-searching, of which seven were already included from the five electronic databases, and therefore did not increase the total number of records imported into Covidence.
